# A role for low-abundance miRNAs in colon cancer: the miR-206/Krüppel-like factor 4 (KLF4) axis

**DOI:** 10.1186/1868-7083-4-16

**Published:** 2012-09-24

**Authors:** Mansi A Parasramka, W Mohaiza Dashwood, Rong Wang, Hassaan H Saeed, David E Williams, Emily Ho, Roderick H Dashwood

**Affiliations:** 1 Linus Pauling Institute, Oregon State University, Corvallis, Oregon, USA; 2 Department of Environmental and Molecular Toxicology, Oregon State University, Corvallis, Oregon, USA; 3 School of Biological and Population Health Sciences, Oregon State University, Corvallis, Oregon, USA; 4 R.H. Dashwood, 479 Linus Pauling Science Center, Oregon State University, Corvallis, OR, 97331, USA

**Keywords:** Cancer stem cells, Colon cancer, Epigenetics, KLF4, microRNAs, miR-206, Pluripotency factors

## Abstract

**Background:**

MicroRNAs (miRNAs or miRs) are short non-coding RNAs that affect the expression of genes involved in normal physiology, but that also become dysregulated in cancer development. In the latter context, studies to date have focused on high-abundance miRNAs and their targets. We hypothesized that among the pool of low-abundance miRNAs are some with the potential to impact crucial oncogenic signaling networks in colon cancer.

**Results:**

Unbiased screening of over 650 miRNAs identified miR-206, a low-abundance miRNA, as the most significantly altered miRNA in carcinogen-induced rat colon tumors. Computational modeling highlighted the stem-cell marker Krüppel-like factor 4 (*KLF4*) as a potential target of miR-206. In a panel of primary human colon cancers, target validation at the mRNA and protein level confirmed a significant inverse relationship between miR-206 and *KLF4*, which was further supported by miR-206 knockdown and ectopic upregulation in human colon cancer cells. Forced expression of miR-206 resulted in significantly increased cell proliferation kinetics, as revealed by real-time monitoring using HCT116 cells.

**Conclusions:**

Evolutionarily conserved high-abundance miRNAs are becoming established as key players in the etiology of human cancers. However, low-abundance miRNAs, such as miR-206, are often among the most significantly upregulated miRNAs relative to their expression in normal non-transformed tissues. Low-abundance miRNAs are worthy of further investigation, because their targets include KLF4 and other pluripotency and cancer stem-cell factors.

## Background

MicroRNAs (miRNAs or miRs) influence multiple stages of cancer development, via post-transcriptional mechanisms that degrade or repress target messenger RNAs (mRNAs) [1]. Several miRNAs with critical roles in early embryonic development [2] become aberrantly expressed in tumors [3]. For example, miR-21 is a high-abundance miRNA upregulated in cancers of the breast, lung, colon, liver, pancreas, prostate, esophagus, brain, and thyroid; targets of miR-21 include phosphatase and tensin homolog, tropomyosin 1, and programmed cell death 4 [4,5].

Evolutionarily conserved high-abundance miRNAs, such as miR-21, have been profiled in various human cancers [6-8], but little is known about the role of low-abundance miRNAs. We hypothesized that certain low-abundance miRNAs might regulate key players in normal physiology such that, under normal circumstances, their expression is tightly restricted.

One such candidate is miR-206. This miRNA has been implicated in breast and lung cancer via the inhibition of notch3 signaling, cell migration, proliferation, metastasis, and invasion [9,10]. Moreover, levels of miR-206 were inversely proportional to c-met expression, an important proto-oncogene in rhabdomyosarcoma [11]. Lin *et al.*[12] identified an autoregulatory feedback loop between miR-206 and Krüppel-like factor 4 (KLF4). This zinc finger protein plays a crucial role in early development and cancer stem-cell biology [13]. KLF4 promotes tumor formation in tissues such as breast and skin [14,15], but suppresses malignancy in cervix, prostate, bladder, esophagus, and colon [16-21]. Loss of the *KLF4* region on chromosome 9q is reported in 25% to 50% of sporadic colorectal cancers, and a significant decrease in KLF4 expression is observed in adenomas and adenocarcinomas of the large and small intestine [22,23].

In a recent investigation of carcinogen-induced rat colon tumors [24], we identified a loss of multiple let-7 family members coinciding with increased expression of miRNA-binding proteins Lin28A/Lin28B, as well as the stem-cell factors c-Myc, Sox2, Oct-3/4, and NANOG. One notable omission from the list of downstream targets was Klf4, a well-established pluripotency factor [13]. Interestingly, among the entire set of 679 miRNAs profiled in rat colon tumors, the greatest relative increase compared with normal tissue was for the low-abundance miR-206. This investigation, therefore, sought to clarify the role of miR-206 and its putative target *KLF4* in colon cancer development. This report provides the first comprehensive analysis of the miR-206/KLF4 axis in parallel studies involving a preclinical colon carcinogenesis model, human primary colon cancers, and a panel of human colon cancer cells.

## Results

### Increased miR-206 and attenuated *Klf4* expression in rat colon tumors

**Figure 1:**
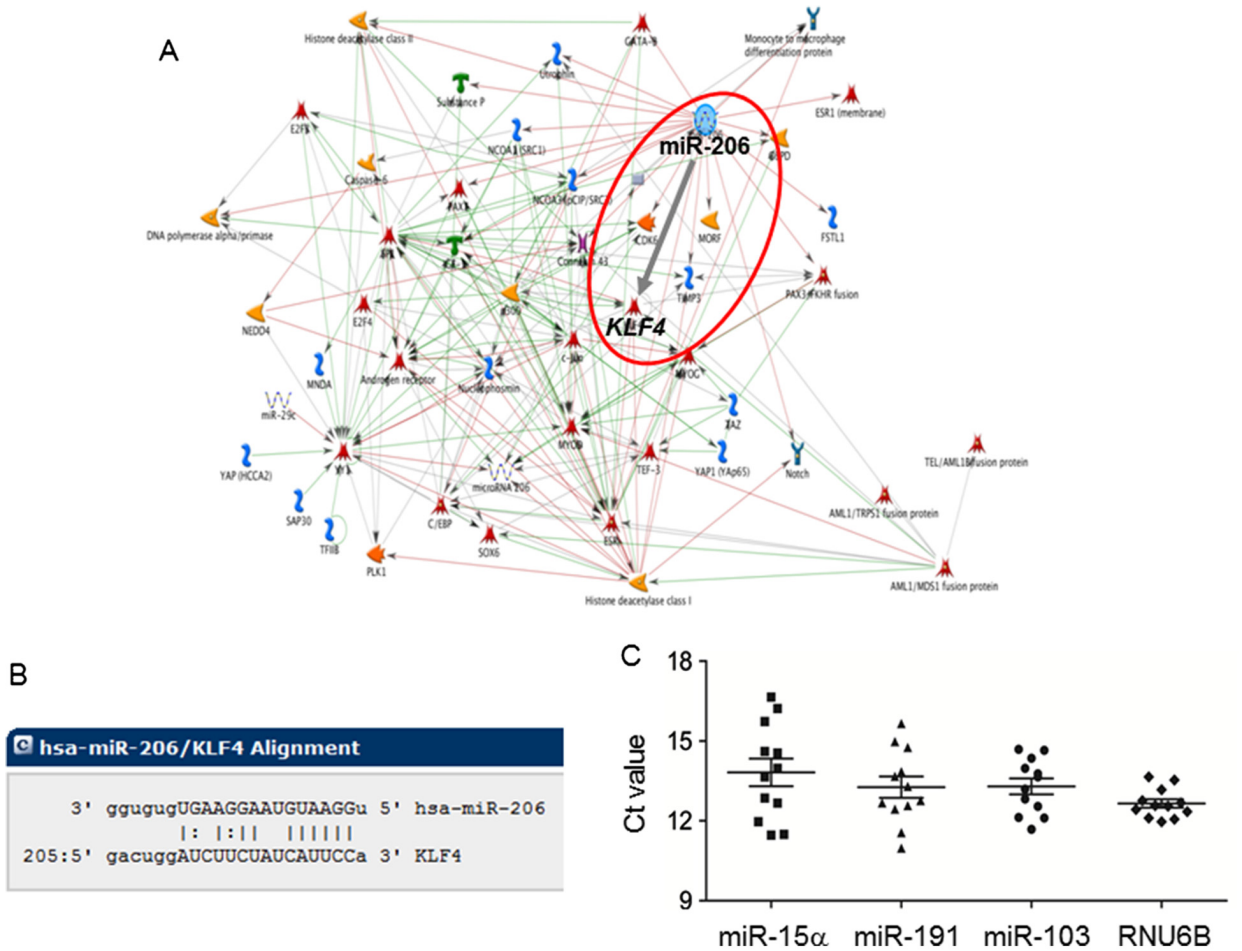
**The miR-206/KLF4 axis in colon cancer.** MicroRNA arrays [22] identified miR-206 as a low-abundance miRNA with potentially important roles in carcinogen-induced rat colon tumors. (**A**) Metacore pathway analysis encompassing rat, mouse, and human miRNAs highlighted *KLF4* as an important target of miR-206, which was supported (**B**) by computational alignment. (**C**) Quantitative real-time PCR (qRT-PCR) assays revealed *RNU6B* as a suitable internal control for subsequent experiments using the rat model, see Figure 2.

Among 679 unique miRNAs profiled, miR-206 was the most dramatically altered miRNA in rat colon tumors, exhibiting ~73-fold higher expression relative to the corresponding normal-looking colonic mucosa (microarray data not shown). Metacore pathway analysis predicted multiple targets of miR-206, including *KLF4* (Figure 1A), which was further supported by sequence complementarity alignment (Figure 1B). To validate these findings, we first screened a number of potential endogenous controls, selecting RNU6B as a suitable candidate for subsequent quantitative real-time polymerase chain reaction (qRT-PCR) assays (Figure 1C).

**Figure 2:**
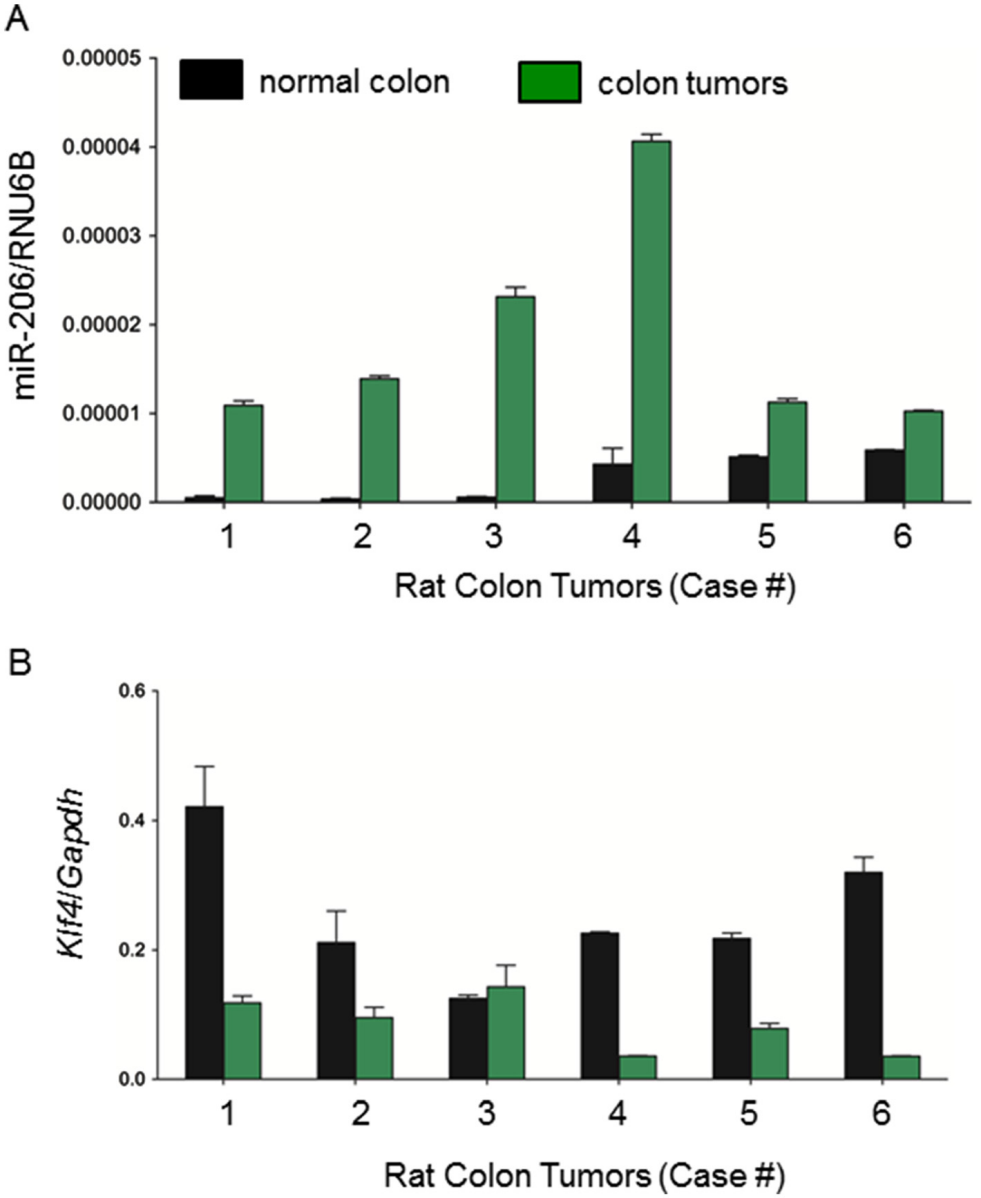
**miR-206 and Klf4 expression in rat colon tumors.** Carcinogen-induced colon tumors and adjacent normal-looking colonic mucosa from a prior study [22] were examined for (**A**) miR-206 expression, normalized to RNU6B, and (**B**) *Klf4* mRNA expression normalized to *Gapdh*. For the six colon tumors shown, data bars indicate mean ± SD, *n* = 3.

In six randomly selected rat colon tumors, miR-206 levels were expressed at higher levels, relative to the corresponding normal-looking colonic mucosa (Figure 2A). These colon tumors had the predicted inverse relationship with *Klf4* mRNA levels, normalized to *Gapdh* (Figure 2B). Specifically, for miR-206 the tumor mean ± SD was 1.8 × 10^−5^ ± 4.5 × 10^−7^ versus matched controls 2.8 × 10^−6^ ± 4.6 × 10^−6^ (*P* < 0.0001, *n* = 6). For *Klf4* the corresponding data were 0.084 ± 0.0075 in tumors versus 0.25 ± 0.034 in normal colon (*P* < 0.001, *n* = 6). However, we did note that one colon tumor with a high relative level of miR-206 did not exhibit a correspondingly reduced level of *Klf4* (compare data in Figures 2A and 2B for Case 3). These data supported a possible role for miR-206 regulating *Klf4* in some but not all rat colon tumors*.*

### An inverse relationship between miR-206 and *KLF4* in human primary colon cancers

**Figure 3:**
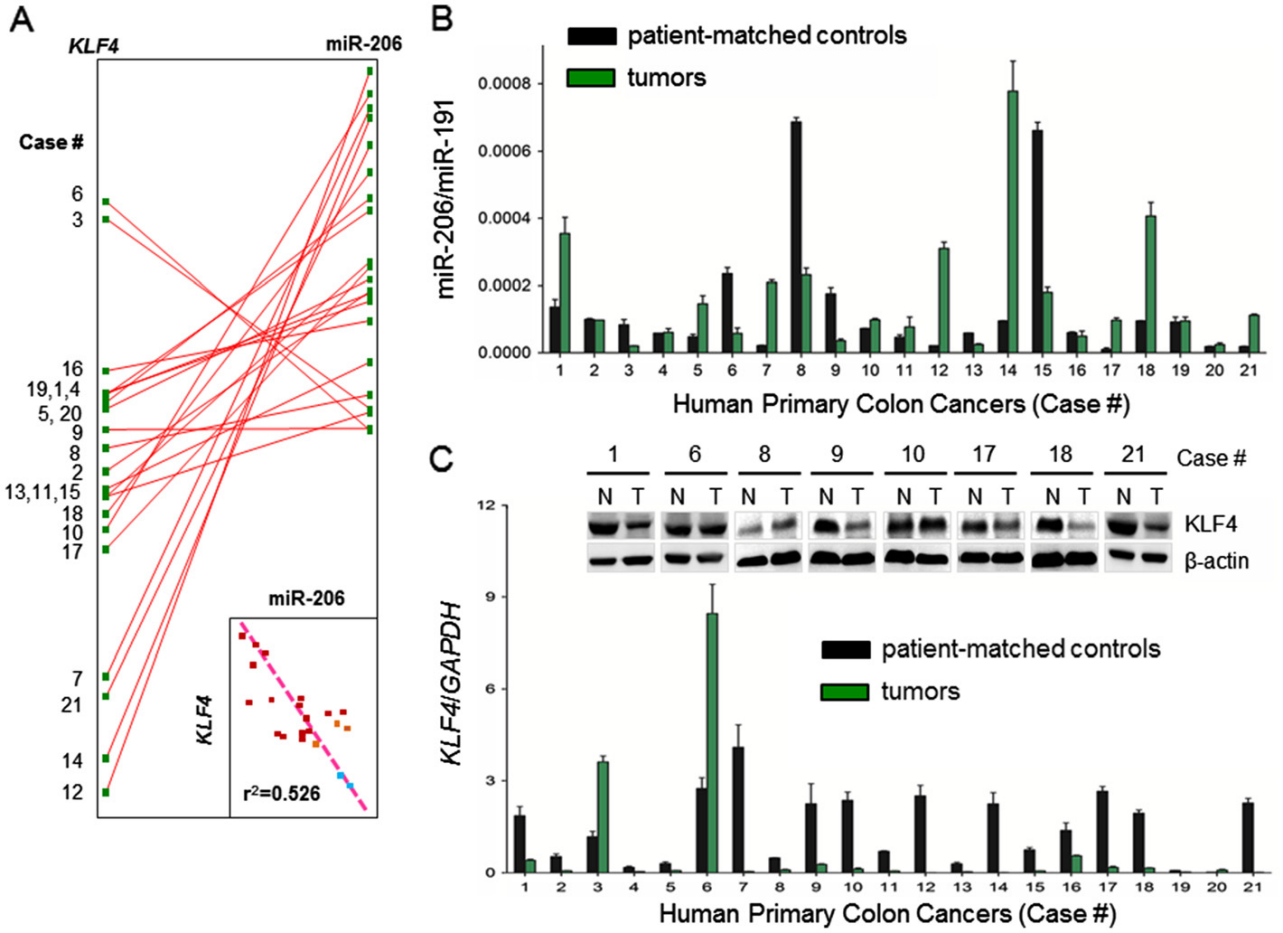
**Inverse association between miR-206 and *KLF4 *in human colon cancers.** (**A**) Twenty-one human primary colon cancers were examined for *KLF4* mRNA expression normalized to *GAPDH*, and for miR-206 normalized to miR-191. Inset: the inverse correlation was significant, *r*^2^ = 0.525, *P* < 0.05. (**B**) Expression of miR-206/miR-191 in 21 primary colon cancers and their patient-matched controls. (**C**) Expression of *KLF4*/*GAPDH* mRNA in the same tumors and matched controls. Representative Western immunoblots are also shown for *KLF4* protein normalized to β-actin.

Based on a prior report [25], human primary colon cancers were first screened for a suitable endogenous control (data not shown); miR-191 was selected for subsequent qRT-PCR analyses. There was an inverse association between miR-206 levels and *KLF4* mRNA expression among the 21 human primary colon cancers examined (Figure 3A, inset, *r*^2^ = 0.525, *P* < 0.05). Several of the clinical cases had higher miR-206 in the cancer compared with the patient-matched control (Figure 3B, green *versus* black bars, respectively); see for example Cases 1, 5, 7, 12, 14, 17, 18, and 21. However, the reverse situation was noted in Cases 6, 8, 9, and 15 (Figure 3B).

Upon further analysis, low *KLF4* mRNA levels were detected in most colon cancers, with the exception of Cases 3 and Case 6 (Figure 3C, normalized to *GAPDH*). Western immunoblotting confirmed the attenuated KLF4 protein level in most, but not all, tumors (T) compared with patient-matched normal (N) tissues (Figure 3C, β-actin loading control). As noted in the rat model, the inverse association between miR-206 and *KLF4* was not always apparent; for example, Case 9 exhibited low relative expression of both miR-206 and *KLF4* in the cancer compared with the patient-matched control (Figure 3B,C). However, inspection of the data in Figures 3B and 3C supported the general hypothesis in 12/21 (57%) of the human primary colon cancers. For miR-206, the tumor mean ± SD was 1.8 × 10^−4^ ± 1.6 × 10^−5^ versus 1.3 × 10 ^−4^ ± 4.6 × 10^−6^ in matched controls (*P* < 0.05). For KLF4, the corresponding data were 0.68 ± 0.0746 in tumors versus 1.467 ± 0.194 in controls (*P* < 0.01).

### Knockdown or ectopic upregulation of miR-206 alters *KLF4* levels in human colon cancer cells

**Figure 4:**
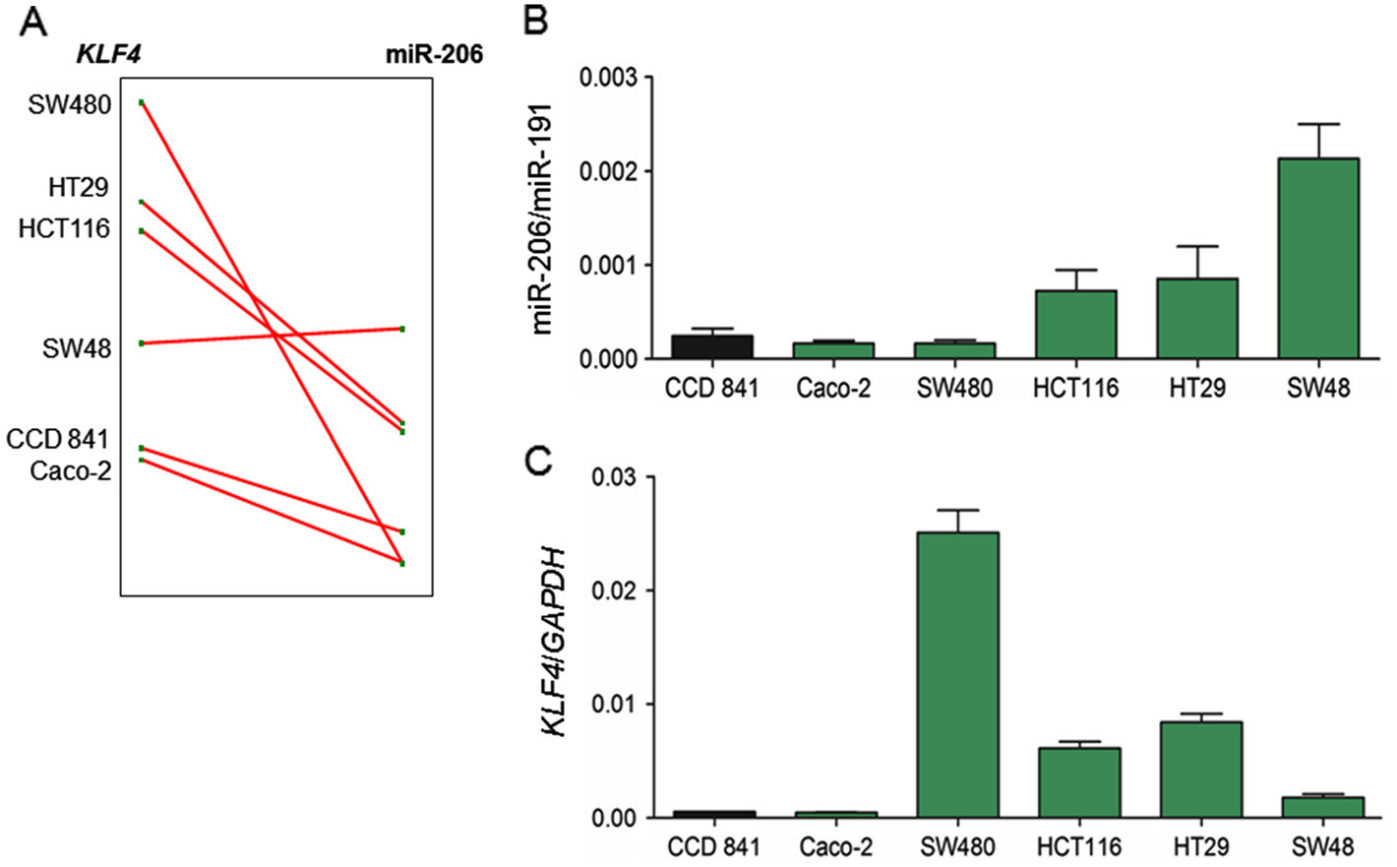
**Constitutive expression of miR-206 and *KLF4 *in human colon cancer cells.** (**A**) Inverse trends for *KLF4* and miR-206 in five colon cancer cell lines and non-transformed colonic epithelial CCD841 cells. (**B**) miR-206/miR-191 and (**C**) *KLF4*/*GAPDH* levels in five colon cancer cell lines (green bars) and CCD841 cells (black bars); data bars indicate mean ± SD, *n* = 3.

In an initial screen of human colon cancer cell lines (Figure 4), SW48 cells had the highest constitutive levels of miR-206 and low relative expression of *KLF4*, whereas SW480 cells exhibited the highest endogenous *KLF4* levels and low miR-206 expression. For the same molecular endpoints, intermediate expression was detected in HCT116 and HT29 cells. Caco-2 cells exhibited low endogenous levels of both miR-206 and *KLF4*, similar to the non-cancer colon embryonic epithelial cell line CCD841 (Figure 4, solid black bar).

**Figure 5:**
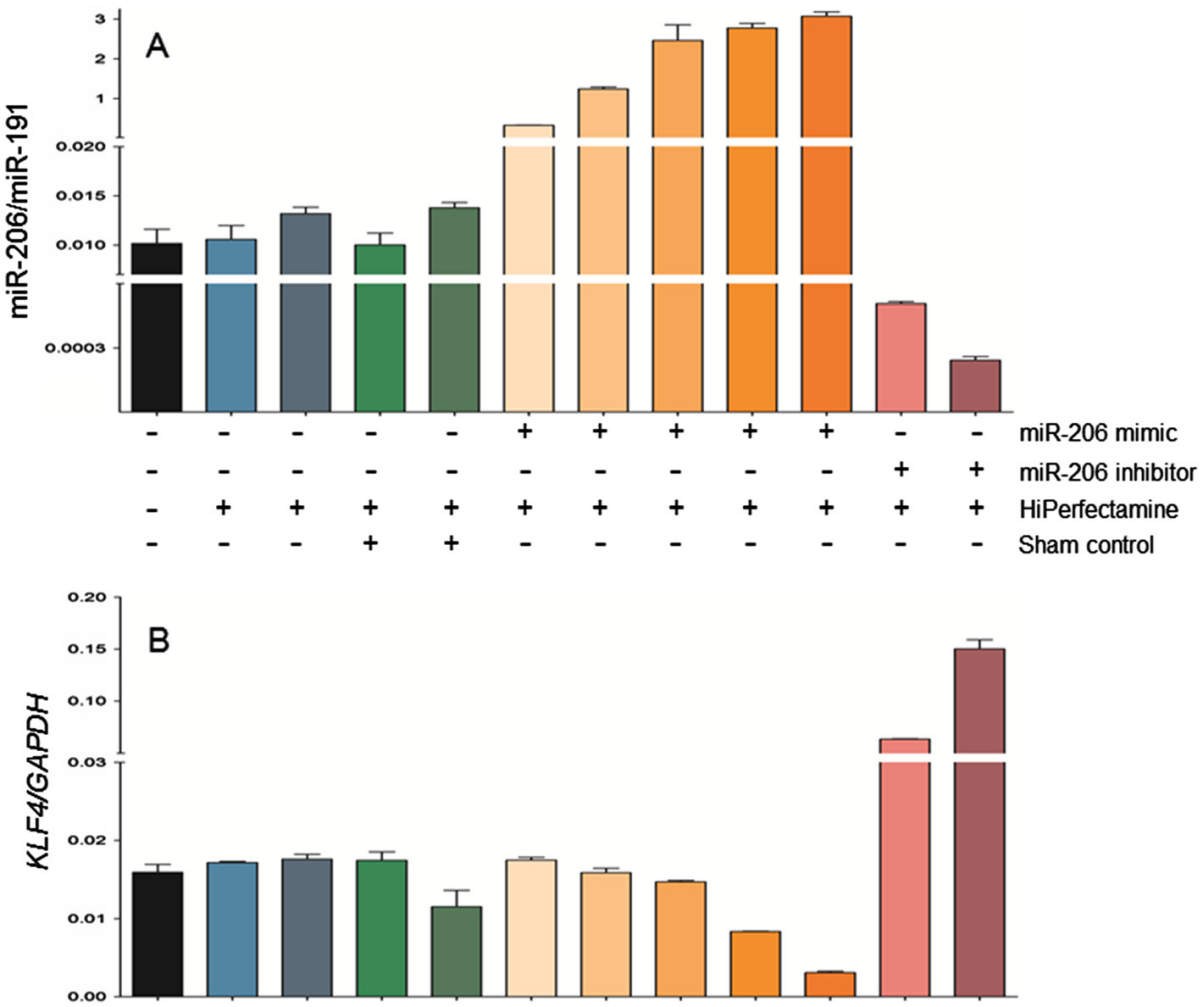
**Knockdown and ectopic upregulation of miR-206 reciprocally regulates *KLF4*.** Human HCT116 colon cancer cells were transfected with increasing concentrations of a miR-206 mimic (graded orange-shaded bars indicating increasing miR-206), or miR-206 inhibitor (pink bars). Black, blue, and green bars designate the various controls, as mentioned in the text. (**A**) miR-206/miR-191 expression and (**B**) *KLF4*/*GAPDH* levels were measured as described for human primary colon cancers, see Figure 3 legend. Data bars = mean ± SD, *n* = 3.

We elected to use an intermediate expressing cell line, HCT116, for subsequent experiments, designed to knockdown or ectopically upregulate miR-206 (Figure 5). A dose-dependent increase in miR-206 expression was observed on transfecting cells with miR-206 mimic (30 to 70 nM), relative to untreated cells (black bar), vehicle treatment (30 and 40 μl HiPerFectamine, blue bars), or sham control (green bars). Under the same conditions, enforced increase of miR-206 resulted in loss of *KLF4* (compare orange bars in Figure 5B with those in Figure 5A). In contrast, HCT116 cells treated with miR-206 inhibitor had a significant reduction in miR-206 expression, and this was associated with a corresponding increase in *KLF4* (compare pink bars in Figure 5A and 5B). These findings supported an inverse association between miR-206 and *KLF4* in colon cancer.

### Enforced increase of miR-206 levels augments proliferation kinetics in human colon cancer cells

**Figure 6:**
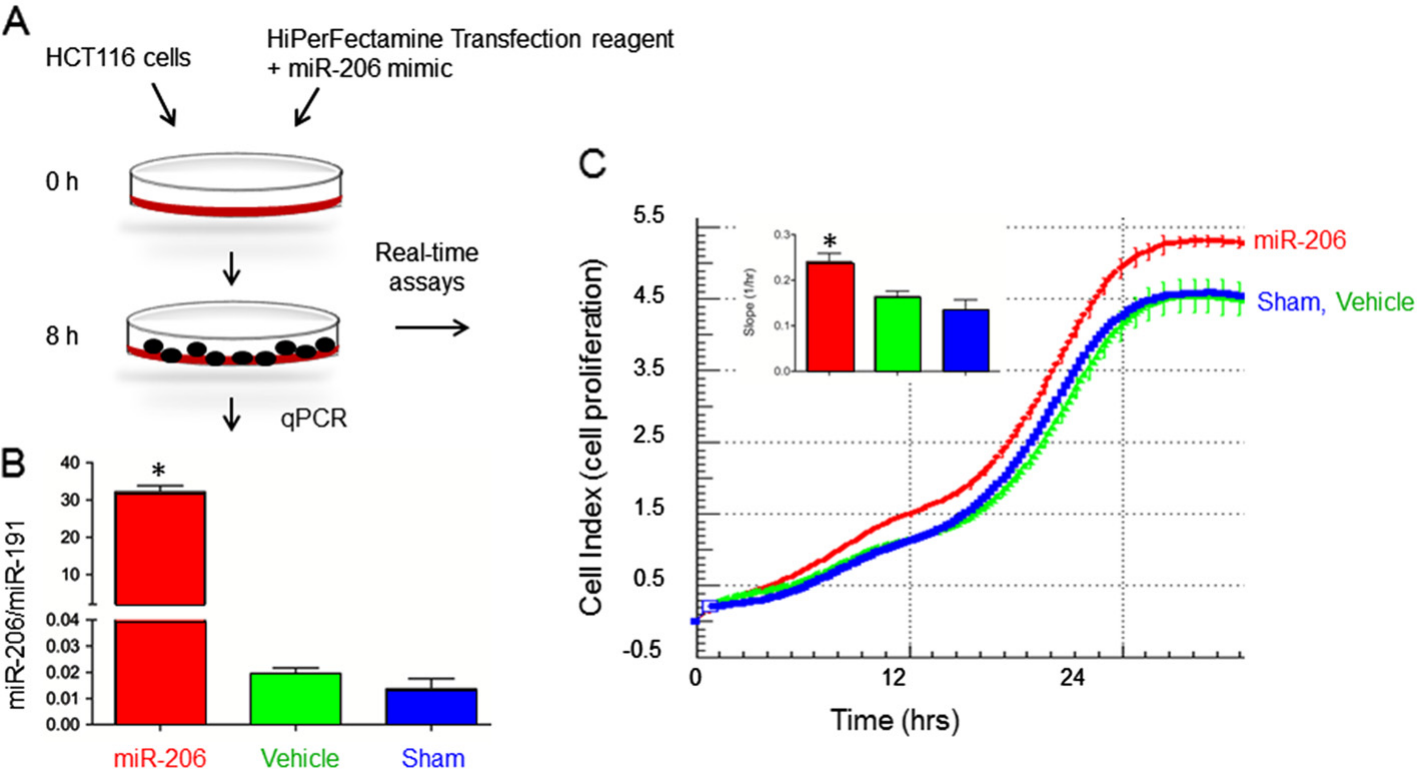
**Cell proliferation is increased by miR-206.** (**A**) Experimental protocol for the treatment of HCT116 cells. (**B**) qRT-PCR data confirmed upregulation of miR-206 expression. (**C**) Real-time monitoring assays using the xCELLigence system [26]. Data bars indicate mean ± SD, *n* = 3.

Eight hours after HCT116 cells were transfected with miR-206 mimic, qRT-PCR assays corroborated the expected increase in miR-206 levels relative to vehicle and sham controls (Figure 6A,B). Reduced *KLF4* expression was also confirmed under the same experimental conditions (data not shown). Cells were loaded onto E-plates (xCELLigence system, Roche) for real-time monitoring assays [26], which revealed a significant increase in cell proliferation kinetics following the enforced increase of miR-206 levels (Figure 6C, inset, *P* < 0.05).

## Discussion

The KLF4 protein contains both activator and repressor domains, and can function as a positive or negative regulator of gene expression [27-29]. Oncogenic effects of KLF4 have been reported in breast, skin, and lung cancers [14,15,30]. However, KLF4 also acts as a tumor suppressor in human colon cancer development [31]. Information on tumor stage was available for some, but not all, of the human primary colon cancers examined here, and as a consequence we could not corroborate an early report suggesting KLF4 as a prognostic predictor of colon cancer [20]. Prior studies in human colon cancer cell lines and primary colorectal cancers implicated hypermethylation of the *KLF4* gene promoter, loss of heterozygosity, or mutation of the open reading frame [17]. Recently, a *KLF4/*miR-206 autoregulatory feedback loop was reported to regulate protein translation reciprocally in normal and cancer cells [12]. In this investigation of rat colon tumors, human primary colon cancers, and human colon cancer cell lines, the data supported an inverse trend between *KLF4* and miR-206.

Located on chromosome 6p12.2, miR-206 is similar in expression and function to miR-1, but its sequence differs by four nucleotides [32]. Studies in breast cancer models have reported tumor suppressive effects of miR-206 due to its pro-apoptotic properties, via the inhibition of notch3 signaling and cell migration [9,33] or proliferation [10]. Furthermore, miR-206 was one of five miRNAs that exhibited stage-dependent differential expression in human colorectal cancers [33]. Interestingly, the latter report noted, in contrast to the current study, that miR-206 levels were more typically attenuated whereas let-7a was increased in the cancers. Given that let-7 family members are normally ascribed a tumor suppressor function, the authors speculated that a high let-7a/low miR-206 ‘signature’ might designate colon tumors with a unique phenotype in terms of cancer progression, compartmentalization, or microenvironment [33]. This subset of cancers would differ from the more typical scenario involving increased miR-206 and reduced let-7a expression.

Loss of let-7a, and of other let-7 family members, coincided with changes in other high-abundance miRNAs in the heterocyclic amine-induced rat colon tumors examined here [24]. Interestingly, however, when the dataset of 679 miRNAs was taken in its entirety, the low-abundance miR-206 was identified as the most significantly upregulated (up to 73-fold) in rat colon tumors relative to normal colonic mucosa. In a separate study using azoxymethane as the initiating agent, miR-206 was also significantly upregulated (~100-fold) in rat colon tumors [34]. The latter report did not pursue miR-206 further, owing to its low abundance relative to other miRNAs. Nonetheless, we were intrigued that two quite different colon carcinogens increased miR-206 so dramatically in colon tumors, relative to normal colonic mucosa.

Thus, we next examined a panel of human primary colon cancers and detected increased miR-206 levels in ~50% of the cases. As predicted by computational modeling, a significant inverse relationship was noted between miR-206 and *KLF4* (Figure 3A, inset). Other known colon cancer miRNA signatures were confirmed, such as an increase in miR-21 and a decrease in miR-98, but no concordance was observed for these miRNAs and *KLF4* (data not shown). To provide further proof-of-concept, we examined a panel of human colon cancer cell lines and noted that SW480 and SW48 cells provided the best evidence for an inverse association between miR-206 and *KLF4*. In cells with intermediate constitutive levels, forced expression or knockdown of miR-206 resulted in the expected reciprocal changes in *KLF4*, and miR-206 ectopic upregulation increased cell proliferation kinetics in real-time monitoring assays. Interestingly, Caco-2 cells resembled non-transformed CCD841 cells, as well as certain human primary colon cancers (Cases 9 and 15, Figure 3), in having low expression levels of both miR-206 and *KLF4*. The latter signature appeared to involve slower cell proliferation and doubling time, although further work is needed to clarify this possibility, especially *in vivo*. Among the other factors that might contribute *in vitro*, miR-206 expression was influenced by cell confluency, being about threefold higher at 70% versus 20% confluency (MAP *et al*., unpublished data).

In contrast to these observations, cancers of the breast and lung have been characterized as exhibiting low miR-206 expression levels, suggesting a possible tumor suppressor function [9,10,12]. Changes in the expression of *KLF4* also can be site specific, being upregulated in cancers of the breast, skin, and lung, but attenuated in colon and gastric tumors [14,15,19,31]. The prior investigation of high-abundance miRNAs in rat colon tumors highlighted a role for c-Myc, Oct-3/4, and Sox2 [24], whereas the present work has implicated a fourth ‘defined factor’ for pluripotency, namely *KLF4*[13]. Further studies are in progress on the role of pluripotency factors, cancer stem markers, and other potential molecular targets of the miR-206/*KLF4* axis (Figure 1).

## Conclusions

This investigation has focused on the possible role of miR-206 and one of its predicted targets, *KLF4*, in colon cancer development. As a low-abundance miRNA, miR-206 might have been overlooked but for the fact that it was the most significantly increased miRNA in rat colon tumors, relative to normal colonic mucosa. An inverse correlation was noted between miR-206 and *KLF4* in a panel of human primary colon cancers, which was supported by experimental studies involving knockdown and ectopic upregulation of miR-206 levels in human colon cancer cells. We conclude that among the low-abundance miRNAs normally ignored or ‘discarded’ during profiling studies there may be key players worthy of mechanistic investigation, depending on the tissue and cancer type.

## Methods

### Cell culture and transfection

Human colorectal cancer lines HCT116, HT29, Caco2, SW48, and SW480 (American Type Culture Collection, Manassas, VA) were maintained in McCoy’s 5A medium (Invitrogen) supplemented with 10% heat-inactivated fetal bovine serum (FBS, Hyclone Laboratories), 100 units/ml penicillin, and 100 μg/ml streptomycin at 37°C in 5% CO_2_. The human embryonal colon epithelial cell line CCD841 was maintained in Eagle’s minimum essential medium (EMEM) according to the recommended ATCC protocol. Each cell line was confirmed independently to be of human origin with no mammalian interspecies contamination, and had the correct genetic profile based on allele-specific markers (Idexx Radil, Columbia, MO).

Transfection of HCT116 cells was performed using a miR-206 mimic (Qiagen, Valencia, CA, USA). Cells were seeded in 100-mm cell-culture grade Petri dishes and transfected at 70% confluency using Qiagen HiPerFectamine Transfection reagent, according to the manufacturer’s protocol. Final concentrations were as follows: miR-206 mimic 30–70 nM, miR-206 inhibitor 50 and 75 nM, AllStars Negative Control 25–75 nM, or HiPerFectamine 30–40 μl in 1000 μl serum-free McCoy’s 5A medium. Untreated cells received complete media only. Cells were harvested after 60 h for determination of miRNA and mRNA expression.

### Rat and human colon tumors

The preclinical study was approved by the Institutional Animal Care and Use Committee (ACUP 3168). Male F344 rats, 3–4 weeks of age, were housed in a ventilated temperature-controlled room at 25°C with a 12 h light and dark cycle. After acclimatization to the basal diet (AIN-93 G), rats were treated by oral gavage with three cycles of a heterocyclic amine carcinogen. Details of the dosing protocol were reported elsewhere [24]. After the last dose of carcinogen, in week 18, rats were switched to AIN-93 M diet until the study was terminated at 52 weeks. Colon tumors and normal-looking colonic mucosa samples were flash-frozen in liquid nitrogen and stored at −80°C prior to miRNA isolation.

Primary human colon cancers and patient-matched controls were provided under an IRB-approved protocol by Steven F. Moss and Lelia Simao (Rhode Island Hospital, Providence, RI). The tumors were characterized as late-stage adenocarcinomas, as reported by Wang *et al*. [26].

### MicroRNA extraction

Cells from experiments conducted *in vitro* were homogenized in ice-cold lysis buffer and flash-frozen. After thawing, cell lysates, human and rat colon tumors, and the corresponding matched controls were homogenized on ice and microRNA isolation was performed using the miRNeasy kit (Qiagen). RNA quality and integrity was evaluated from the absorbance at 260 and 280 nm (260/280 ratio > 1.9).

### MicroRNA microarrays and computational analyses

Microarray analyses of 679 unique mature target rat miRNAs (from miRBase version 17, http://miRBase.org) were performed by LC Sciences (Houston, TX), as detailed elsewhere [24]. Images were collected using a GenePix 4000B scanner (Molecular Devices, Sunnyvale, CA) and digitized using Array-Pro image analysis software (Media Cybernetics, Bethesda, MD). Data were analyzed by first subtracting the background and then normalizing the signal using a locally weighted regression filter. Cluster plots were generated using software from The Institute for Genomic Research.

For human primary colon cancers and their matched controls, miR-206 and *KLF4* expression values were analyzed by Benjamini-Hochberg correction using ArrayStar software (DNASTAR, Inc., Madison, WI, USA). Scatter plots were evaluated by simple linear regression analysis. Interaction between the expression profiles among the samples was compared by hierarchical cluster analyses and line graph using ArrayStar software. A difference with *P* < 0.05 was considered statistically significant.

### Biological network analyses

Putative target mRNAs of miR-206 were examined via MetaCore pathway analysis (GeneGo Inc., St Joseph, MI, USA). For pathway enrichment analysis, *P* values were calculated using the formula for hypergeometric distribution, reflecting the probability of a pathway arising by chance. Pathway maps were prioritized based on statistical significance.

### Validation of microRNA expression

The qRT-PCR analyses used the Qiagen miScript kit according to the manufacturer’s instructions, with miR-206-specific primers. RNA (~1 μg) from colon tumors or normal controls was reverse-transcribed, and qRT-PCR was performed in a 20 μl reaction containing miScript Universal primer, miRNA specific-miScript primers, SYBR green mix, and template cDNA. Reactions were conducted in triplicate and fluorescence intensities were acquired using a LightCycler 480 II (Roche Applied Science, Indianapolis, IN, USA). Relative miRNA expression was quantified by determining the point at which the fluorescence accumulation entered the exponential phase (Ct), and the Ct ratio of the target was normalized to small nuclear RNA U6B (RNU6B) or miR-191.

### Messenger RNA expression analysis

Target mRNAs were quantified by qRT-PCR and normalized to *glyceraldehyde-3-phosphate dehydrogenase* (human *GAPDH*, rat *Gapdh*). Briefly, 1 μg total RNA was reverse-transcribed using SuperScript III First Strand Synthesis Supermix Kit (Invitrogen, Eugene, OR, USA), and qRT-PCR was conducted in a 20 μl reaction containing cDNAs, SYBR Green I dye, and primers *KLF4*-F: CCAATTACCCATCCTTCCTG, *KLF4-*R: CGATCGTCTTCCCCTCTTTG (human) or *Klf4*–F: CAGACCTGGAAAGTGGTGG, *Klf4*-R: ACCTGTGTTGCCCGCAGCC (rat). Experiments were conducted in a Light Cycler 480 II, and the Ct ratio of the target gene to *GAPDH* or *Gapdh* was calculated. Three separate experiments were performed in triplicate, for each sample.

### Western immunoblotting

KLF4 protein levels were examined using the Western immunoblotting methodology reported elsewhere, with β-actin as loading control [24]. The primary antibody was rabbit polyclonal KLF4 lot no. GR45275-3 (Abcam, Cambridge, MA, USA), at 1:500 dilution.

### Cell proliferation assays

Real-time cell proliferation assays were conducted using the xCELLigence System (Roche). HCT116 human colon cancer cells were transfected with miR-206 mimic, as described previously. Cells transfected with mimic, vehicle (HiPerFectamine), or sham negative control were harvested at 8 h. Cells (1.5 × 10^3^) in 100 μl serum-free media were loaded onto the pre-soaked Roche E-plates and measurements were taken at intervals of 10 min [26].

### Statistics

Data were plotted as mean ± SD and compared using one-way and two-way ANOVA (Graphpad Prism 5 software). In the figures, significant outcomes were shown as: *P < 0.05, **P < 0.01, ***P < 0.001.

## Abbreviations

ANOVA: analysis of variance; EMEM: Eagle’s minimum essential medium; KLF4: Krüppel-like factor 4; *KLF4*: Human gene coding for KLF4; *Klf4*: Rat gene coding for Klf4 protein; miRNA or miR: microRNA; mRNA: messenger RNA; qRT-PCR: quantitative real-time polymerase chain reaction.

## Competing interests

The authors declare no conflict of interest.

## Authors’ contributions

MAP conducted the molecular studies, immunoassays, and computational analyses, and drafted the manuscript. WMD and RW oversaw the one-year carcinogenicity bioassay. HS participated in immunoassays, computational analyses, and miRNA isolation. DEW and EH participated in the experimental design, and helped to draft the manuscript. RHD conceived the study, supervised the work, and was responsible for manuscript edits. All authors read and approved the final version of the manuscript.
